# High detail resolution cellulose structures through electroprinting

**DOI:** 10.1038/s41598-024-78526-9

**Published:** 2024-11-12

**Authors:** Farnaz Rezaei, Daniel O. Carlsson, Jimmy Hedin Dahlstrom, Jonas Lindh, Stefan Johansson

**Affiliations:** 1 Department of Materials Science and Engineering, Uppsala University, 75105 Uppsala, Sweden; 2 Cytiva, Björkgatan 30, 753 23 Uppsala, Sweden

**Keywords:** High-resolution 3D printing, Electroprinting, Cellulose acetate, Additive manufacturing, Techniques and instrumentation, Bioinspired materials

## Abstract

Electrospinning is a technique used to fabricate polymer fibers in micro- and nanoscales. Due to the large distance between the nozzle and collector, there is a limited positioning accuracy of electrospun fibers. To enhance the possibility of fabricating structures with micrometer placement, an electroprinting technique has been developed. By reducing the distance between the nozzle and the collector it is demonstrated that it is possible to get an improved control over fiber positioning which gives a possibility to fabricate designed 3D structures at the micron scale. In this study, cellulose acetate (CA) has been selected as a biomaterial to advance the 3D printing of membranes with possible use in separation applications. Various parameters, such as CA concentration and molecular weight, printing speed, printing pattern, applied voltage, etc. are evaluated with respect to printing control. Results indicate that by optimizing the printing parameters it is possible to print structures with inter- fiber distances down to 3 µm and fiber diameters at a sub-µm scale. This electroprinting development is promising for the fabrication of customized separation membranes. However, printing speed still remains a challenge.

## Introduction

Fabrication of diverse ultrathin polymer fibers in micro and nanoscale is possible through the electrospinning technique^1^. Electrospinning allows simple and direct fabrication of nanostructured materials such as nanofibers^2^. Over the last two decades, electrospinning has proven to be practical, effective, and feasible in a wide range of applications^3^. Electrospinning is a technique that uses electric field to form fibers from liquid polymer solution^4^. In typical electrospinning, the polymer solution is initially fed through a spinneret and high voltage is applied between spinneret and collector^5,6^. The solution, which gets charged, is ejected from the spinneret or needle tip when the electric field is sufficient to overcome the surface tension and, after solvent evaporation, the polymeric fibers are gathered on the desired collector^1,7^. Electrospun fibers typically have diameters in the µm or sub-µm region and because of the resulting high surface area, they can be used for various applications, e.g., wound dressing materials, bio-scaffolding, filtration, and separation membranes^7–9^. Membranes, due to their unique advantages such as high efficiency in separation, low cost, and low energy consumption has lately attracted attention^10,11^. In a traditional electrospinning setup, the electrospun fibers have limited positioning accuracy^12^, and fabricating structures with micro and nano placement resolution is not possible. Fiber positioning would be of great value to produce designed structures in several of the mentioned applications as well as in various other contexts^8,13^. To overcome this issue, various researchers have developed a range of techniques to fabricate 3D structures with customized pore size^14^.

Additive manufacturing, or 3D printing techniques, typically based on layer-by-layer material deposition can give the possibility to fabricate arbitrary structures based on the digital designed model^15–18^In electroprinting, also called near-field electrospinning (NFES) or electrohydrodynamic 3-D printing (EHD)^9^, the reduced distance (hundreds of µm) between the nozzle and the collector compared to traditional electrospinning gives the possibility of fabricating more precise structures^11,12^. This technology allows the shift from randomly oriented fibers to precise, well-defined patterns^19^. Using reduced working distance in the electroprinting technique has various advantages, e.g. improved control of the fiber position, reduced voltages, and the possibility of fabricating 3D structures layer by layer^10^. Essential components of electroprinting are a fluid supply system, positioners, a high-voltage power supplier, and a visualizing device^16^. Control over the fiber size, shape, and orientation is achieved by changing and optimizing a range of parameters e.g. nozzle diameter, the movement speed of the collector in different directions, strength of the applied electric field^20,21^, and viscosity of the ink. It should be realized that developing an ink that can easily be extruded from a narrow nozzle and still form a self-supporting 3D-printed feature is a challenge^22^. EHD technique allows for the use of a wide range of materials, such as polycaprolactone (PCL), poly(methyl methacrylate) (PMMA), poly(vinyl cinnamate) (PVCi), and poly(hydroxyl butylate) (PHB), with the printing resolution depending on the chosen parameters such as polymer concentration, printing speed, ect. For instance, using PCL resulted in a printing resolution of 600 µm^23^, and using Polytetrafluoroethylene** (**PTFE) resulted in printing pore sizes with openings of approximately 100 µm^19^. In^18^, an electrostatic technique is used to improve lateral placement control of fibers and the best pitch reached, in the order of 50–75 µm, demonstrates previous placement control for arbitrarily placed fibers.

Cellulose acetate (CA) is derived from cellulose which is an abundant natural polymer^24^and attracts attention due to, for instance, its biodegradable and sustainable nature. Further, the compatibility with the human body, and chemical affinity to various biological components, position CA as a biomaterial of great interest in diverse applications^25^. Among the applications of CA, immobilizing bioactive substances, skin protection, biosensors, bio-separation, and affinity purification membranes are notable^26–28^.

Improving printing precision is crucial for creating more diverse structures with improved properties^29^. This study focuses on cellulose acetate as a biomaterial for fabricating e.g. 3D-printed separation membranes. The aim is to explore the feasibility of electroprinting high-detail resolution structures by optimizing various parameters, e.g. CA concentration, CA molecular weight, printing speed, printing pattern, applied voltage and nozzle size. The results demonstrate the ability to print fibers with an inter-fiber distance within the 3 µm range and fiber diameter in the sub-µm range. Additionally, the possibility of printing free-hanging fibers is demonstrated. The particular circumstances for 3D-electroprinting µm-sized fibers are elucidated, and the feasibility of printing with high placement control is discussed.

## Experimental

### Materials

Cellulose acetate (molecular weights: 30 and 50 kDa), acetone (99.9% purity), and Dimethyl sulfoxide (DMSO 99.5% purity) were purchased from Sigma–Aldrich and used as received.

### Ink preparation

In this study, inks with different amounts of CA (10, 12.5 and 15 weight percent) with molecular weights (MW) of 30 and 50 kDa were examined. CA solutions were prepared using acetone and DMSO with a 1:1 ratio as solvent. All the solutions were mixed with a shaker (Heidolph) for 24 h at 2000 RPM.

### EHD printing setup

The EHD printing setup in this study has been built in-house previously. The setup consists of a high-voltage generator, a syringe pump and a positioning table holding the substrate^30^. The substrate and the nozzle are connected to a high-voltage supply, facilitating the required electric field for EHD printing. The syringes are filled with the CA solutions and connected to the syringe pump, to extrude the ink through a glass capillary with a few µm opening size. The capillaries are pulled with a (P-1000, Sutter instruments) micropipette puller, thereafter shaped and resized with a microforge (Narishige MF-900) enabling control of the size of the nozzle. The positioning table gives precise control (position sensors with 10 nm resolution and position accuracy of about 1 µm) of the substrate in X, Y, and Z directions according to the designed pattern. The chosen substrates were 1 × 1 cm glass slides, coated with Au/Pd (Polaron sputter) on both sides to make them conductive. To be able to initiate and check the printing process, a camera with ×150 magnification is positioned in front of the setup. Nozzles with internal sizes of 3 µm and external sizes of 9 µm were used, unless mentioned otherwise.

### Printing patterns

**Fig. 1:**
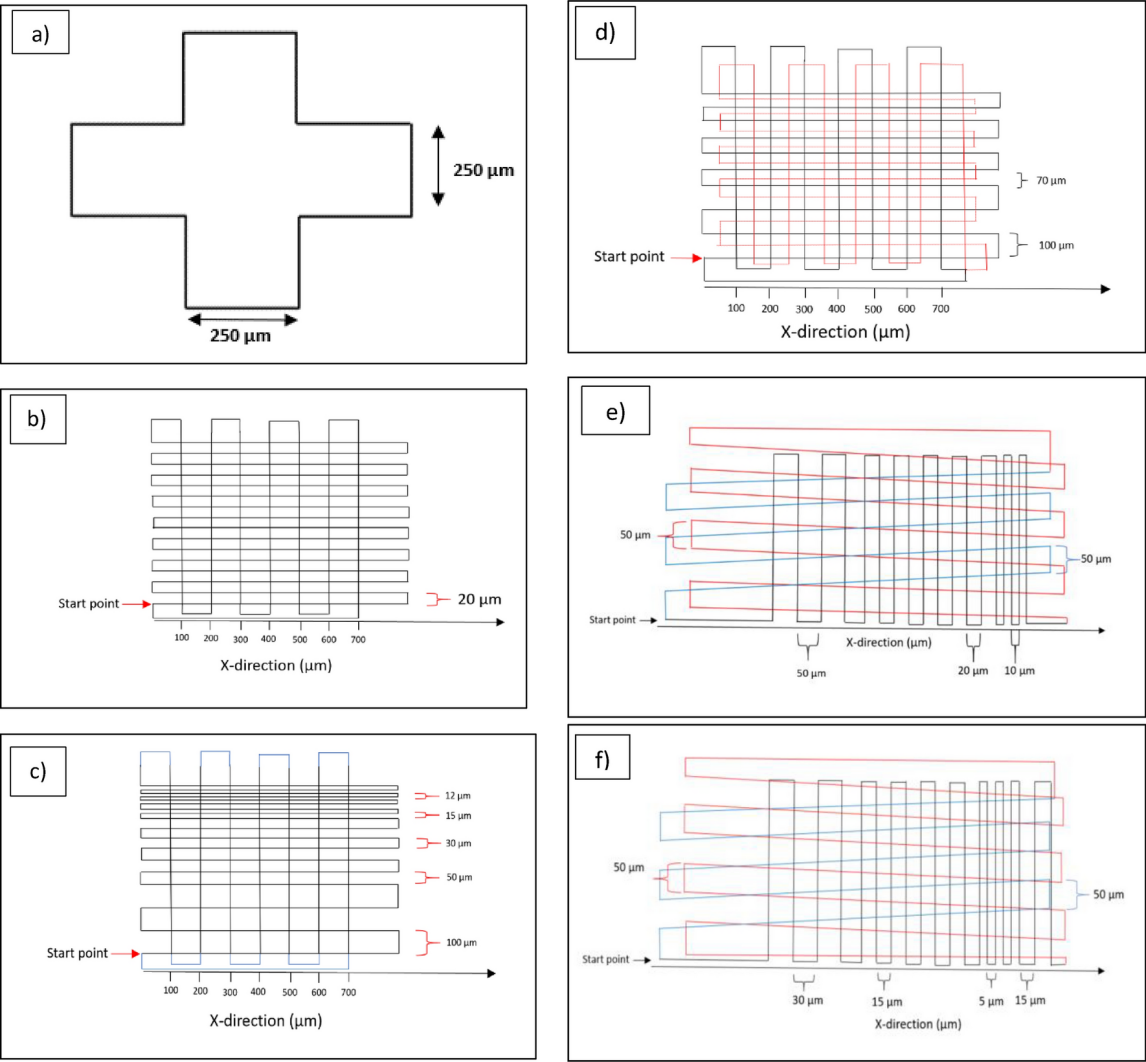
(**a**) Cross shape structure (**b**) Rectangular structure with parallel lines and constant pitches of 100 µm and 20 µm in X and Y directions (**c**) Rectangular structure with parallel lines and different pitches from 100 µm to 12 µm in the X direction and constant pitches of 100 µm in the Y direction (**d**) Rectangular structure with parallel lines, with pitches of 100 µm and 70 µm in the X direction and constant pitches of 100 µm in the Y direction, and shifts between the layers. The first five layers are shown with black lines, while the subsequent five layers have a shift (50 µm and 35 µm in the X direction and 50 µm in the Y direction), which is shown with red lines. (**e**, **f**) The first layer with different pitches from 30 µm to 5 µm indicated with black lines, the second layer has 50 µm pitches and is shown with red lines, and the third layer is shown with blue lines and 50 µm pitches.

To test the printability of the different inks and the possibility of printing with high detail resolution, different structures were printed. As shown in Fig. 1(a), cross-shaped structures were printed as one of the evaluation structures. The second pattern, Fig. 1(b), has parallel lines with a constant pitch of 20 and 100 µm in the Y and X directions, respectively. For some tests, this constant pitch between lines changed to 50 µm in both directions. In a third pattern, which is shown in Fig. 1(c), the pitch in the X direction is constant at 100 µm and the pitch in the Y direction decreases from 100 µm to 50, 30, 15, and 12 µm. In the pattern in Fig. 1(d), the initial five layers are with 100 µm and 70 µm pitches in the Y direction and 100 µm pitches in the X direction (indicated by black lines), and the subsequent three to five layers (red lines) were shifted in both X and Y directions in relation to the first layers, the shift in X direction remains constant at 50 µm, while in the Y direction shifts of 50 µm, 35 µm were made to print in the middle of the already printed black lines. After printing each layer, the substrate moves 1 µm in the Z direction. In Fig. 1(e, f), pitches vary in the X direction. In Fig. 1(e) a first layer starts with pitches from 50 µm and reduces to 20 µm and 10 µm, while in Fig. 1(f) pattern pitches in the X-direction start from 30 µm, decrease to 15 µm and 5 µm, and again increase to 15 µm, shown with black lines. The second layer of the structures (Fig. 1(e, f)) (shown with red lines) is not perpendicular to the first layer and it is tilted relative to the X-axis. Blue lines indicate the third layer of the structure, and it is also tilted relative to the X-axis but opposite to the second layer. Pitches in the second and third layers are constant and equal to 50 µm. After printing each layer substrate moves 1 µm in the Z direction. For printing structures with more than three layers, the pattern repeats cyclically (the fourth layer is the same as the first layer, the fifth layer mirrors the second layer and this repetition continues for subsequent layers).

### Characterization

Ink viscosities were studied with a rotational rheometer (Physica MCR 300, Anton Paar). Viscosity measurements were conducted at 20 °C with varying shear rates (50–1000 s^-1^), and the viscosity values were recorded accordingly. The morphology of the 3D printed membranes was studied by SEM (LEO 1550, Zeiss) after coating the structures with gold and palladium using a Polaron sputter. SEM was performed using in-lens and secondary electron modes.

## Results and Discussion

### Voltage analysis

**Fig. 2:**
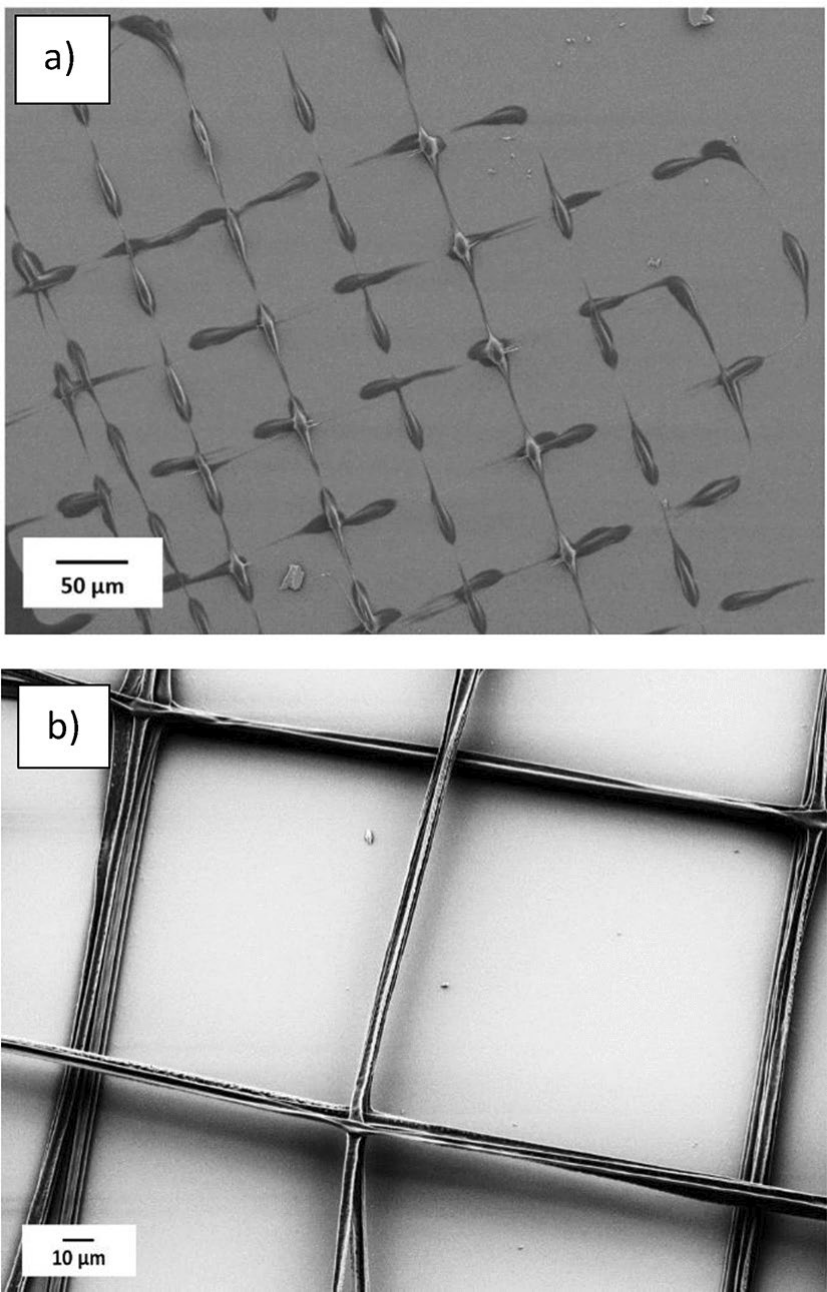
Structure in Fig. 1(d) printed with ink with 12.5% CA (50 kDa) and an applied electrical voltage of: (**a**) 500 V, (**b**) 1000 V.

The dependence on applied voltage in the printing process was studied. The experiments aimed to identify suitable voltage ranges for the different inks to get a stable Taylor cone jet. Electric voltages from 200 to 1500 V were applied to the inks with different compositions with a 10 µm distance between nozzle and substrate and the voltage range resulting in stable cone jetting was identified. Voltages lower than 200 V were excluded for all inks as they did not result in any Taylor cone. At voltages over 1500 V multi-jet printing was observed in all cases, as well as a lack of control over ink extrusion and positioning. To select a suitable voltage, the pattern in Fig. 1 (d) was printed. As is shown in Fig. 2(a) low electric voltage results in non-continuous fibers due to intermittent jetting, here exemplified with 12.5% 50 kDa CA. In this test, the applied electric voltage between the nozzle and the substrate was 500 V. When the electric field was increased to 700–1000 V, stable Taylor cone jetting was obtained. Figure 2(b) shows continuous fibers with uniform width printed at an electric field of 1000 V using the same ink. However, it was challenging to control the size of the printed fibers with inks containing a lower amount of CA. At a concentration of 10% CA (30, 50 kDa), it was difficult to get controlled printing. Despite applying an electric voltage as low as 200 V, there was excessive ink ejection from the nozzle and the printed fibers became much larger than the outer diameter of the nozzle. At the higher amount of CA, 15%, clogging within the capillary occurred regularly. Still, it was possible to print a few layers with an ink consisting of 15% CA (30 kDa), and here the applied voltage was in the range of 1000–1500 V.

### Ink analysis

The printability of inks with six different compositions and the possibility to control the cross-section size of the printed fibers were examined. The outcomes, codified based on colour, are presented in Table 1, providing a relation between voltage, ink composition, and printability.

**Table 1:**
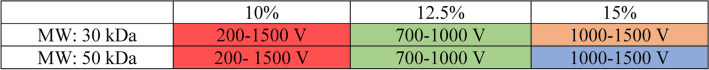
Printability of inks with different amounts of CA and different MW color-coded.

Red: reduced detail resolution with rarely clogging, green: high detail resolution with rarely clogging, orange: high detail resolution with seldom clogging, blue: high detailed resolution with frequent clogging.

As presented in the voltage analysis section, structures were successfully printed using inks containing 12.5% CA (50 kDa) with a voltage in the range of 700-1000 V.

**Fig. 3:**
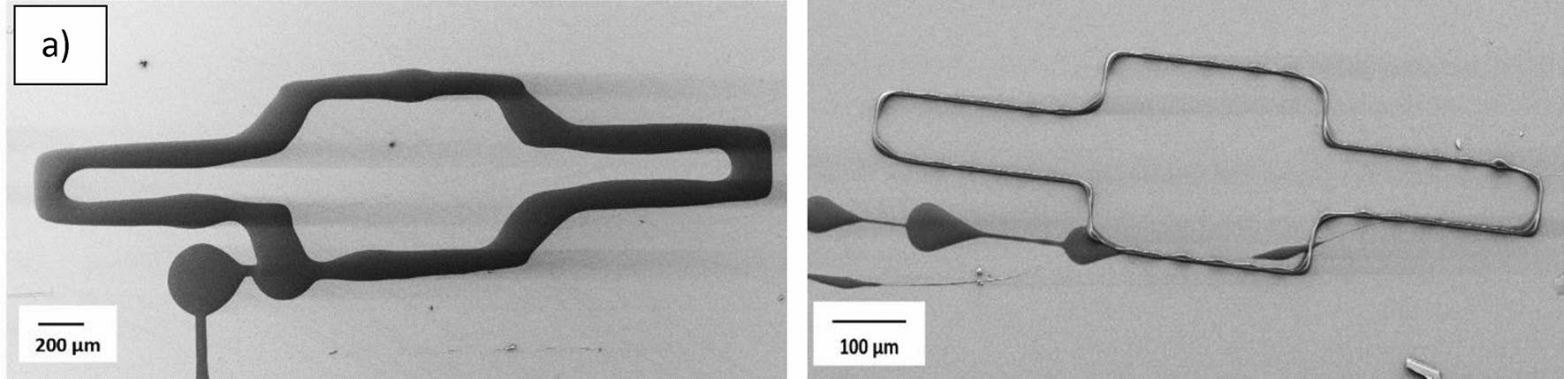
Test structure in Fig. 1(a) printed with applied voltage range 700- 1000 V. Inks for printing structures contain (**a**) 12.5% CA (30 kDa), (**b**) 12.5% CA (50 kDa).

Regarding the molecular weight (MW), results indicate that the controllability of the ink containing 12.5% CA (50 kDa) is higher compared to the ink with lower molecular weight (30 kDa), Fig. 3(a, b). Additionally, the risk of clogging the nozzle is lower using an ink with 12.5% CA (30 kDa) compared to 15% CA (50 kDa). For the following experiments, the ink with 12.5% CA (50 kDa) was selected.

**Fig. 4:**
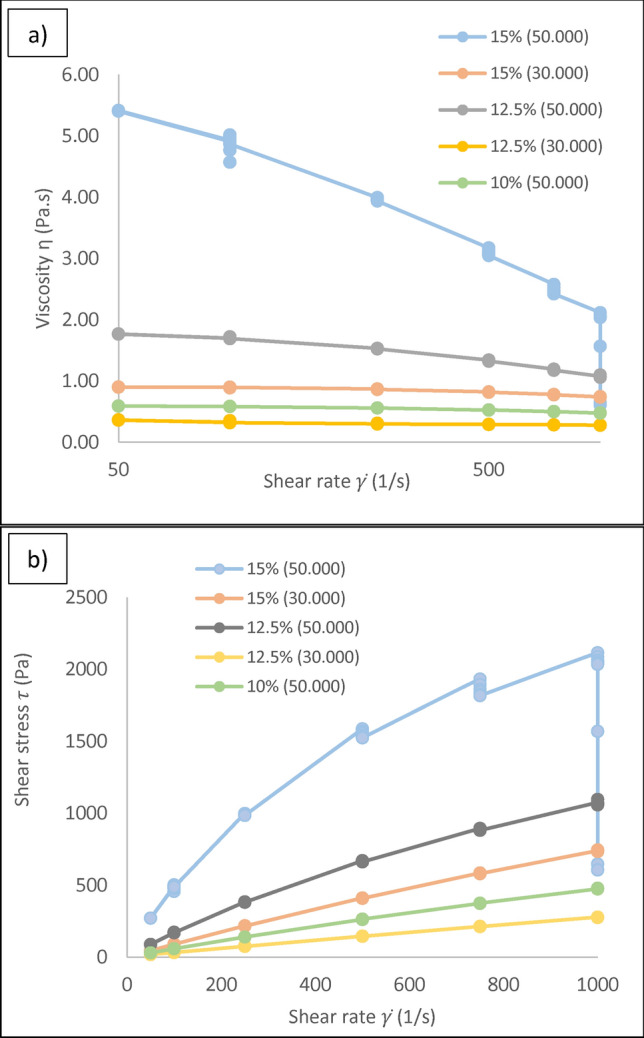
(**a**) Viscosity of the inks as a function of shear rate. (**b**) Shear stress of the ink as a function of shear rate.

The shear thinning behaviour of the ink in extrusion 3D printing is often one of the key parameters. As the ink undergoes shearing during the extrusion process, the viscosity of the ink reduces through the nozzle and returns to higher viscosity after exiting the nozzle. In this study, to clarify the viscosity of the selected inks (orange and green colours in Table 1), measurements with a rheometer were carried out. As shown in Fig. 4(a), a plot of viscosity versus shear rate indicates that the rheological properties of the inks vary with CA concentration and molecular weight. Using the power law equation, $\tau =\text{K}{ {(\gamma )}}^{n}$ where *τ*, is shear stress, K is consistency index, $\dot{\gamma }$ is shear rate and n is power law index, plots of shear stress versus shear rate were generated Fig. 4(b) and the values of power law index (n) were calculated, Table 2.

As shown in Table 2, all the inks in this experiment show shear thinning behaviour. Ink with 15% CA (50 KDa) has the lowest power law index value (0.6) compared to other inks and shows the strongest shear thinning characteristics. Results show that by reducing the molecular weight of CA from 50 KDa to 30 kDa, at the same concentration of 15%, n increases from 0.6 to 0.93 and the shear thinning behaviour of the ink reduces. Inks with a MW of 30 kDa or low concentration of CA exhibit larger power law index values, (compared to the inks with higher amount of CA and molecular weights), which indicates a shift towards Newtonian behaviour (n < 1: shear thinning, n > 1: shear thickening, and n = 1: Newtonian behaviour.). As mentioned in Table 1, due to the frequent clogging of the ink with 15% CA (50 kDa), the ink with 12.5% CA (50 kDa) was selected for the print studies.

**Table 2:** Values of power law index of inks with different amounts of CA and MW.

Ink	Power law index
15% CA (50 kDa)	0.60
15% CA (30 kDa)	0.93
12.5% CA (50 kDa)	0.82
12.5% CA (30 kDa)	0.91
10% CA (50 kDa)	0.92

It should also be noted that apart from the increase in viscosity, due to a reduced shear rate after the ink leaves the nozzle, the fiber solidification will also depend on the loss of solvent. Acetone with its high evaporation rate will leave the ink first resulting in a rapid increase in viscosity and solidification. The speed of evaporation is higher for the finer fibers since the area to volume ratio increase with fiberdiameter.

### Ink flow

The ink is delivered with the help of a syringe pump through a narrow capillary. The narrow capillary is expected to correspond to a noticeable capillary pressure in the order of 1 bar^31^. The movement of the syringe’s piston gives a pressure in the ink to overcome the capillary pressure and in the experiments, the flow rate is adjusted until there is ink flowing through the nozzle. It is observed that the flow rate through the nozzle does not change substantially during a printing session when the syringe pump speed is varied within a rather large range (from zero speed to two times the selected speed). It has proven difficult to eliminate all minute gas bubbles in the syringe and the gas will make it difficult to control the flow with the syringe pump. Considering these observations, to have a constant flow rate through the nozzle, the syringe pump movement speed is kept at the same value, or the pump is turned off when the ink flow through the nozzle begins. The molecular entanglement is also another effect that has to be considered. During printing the ejected fiber will add a force pulling out ink from the nozzle.

### Controlled placement of the fibers

**Fig. 5:**
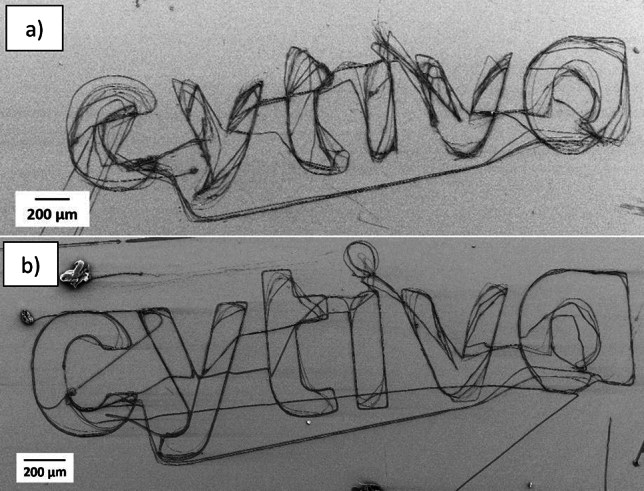
Both patterns were printed with the ink containing 12.5% CA (50 kDa) with an applied voltage of 700–1000 V. The distance between the nozzle and substrate is 40 µm (**a**), and 10 µm (**b**).

It has been observed that the placement of the printed fibers improves with a reduced distance between the nozzle and substrate and the typical minimum distance has previously been a few hundred micrometers^4,14^. In this study the initial distance between the nozzle and the substrate was minimized to 10 µm (the nozzle was withdrawn 10 µm orthogonally from the substrate surface after being in contact with the substrate), to have the nozzle as close to the substrate as possible and still being able to form a Taylor cone. To examine the possibility of printing arbitrary structures and to investigate the impact of the reducing distance between the nozzle and substrate, the name of the company, Cytiva, was printed. In Fig. 5(a), the distance between the nozzle and substrate was 40 µm, while in Fig. 5(b) the distance is reduced to 10 µm. In both structures, 15 layers were printed and the layer thickness was 1 µm with fiber diameters down to sub-micrometer range. Results indicate that a reduced distance provides greater control over the positioning of the fibers and enhanced resolution in the printed structure. As the printing was continuous, some fibers were printed during transition from one part of the structure to another part.

### Printing speed

**Fig. 6:**
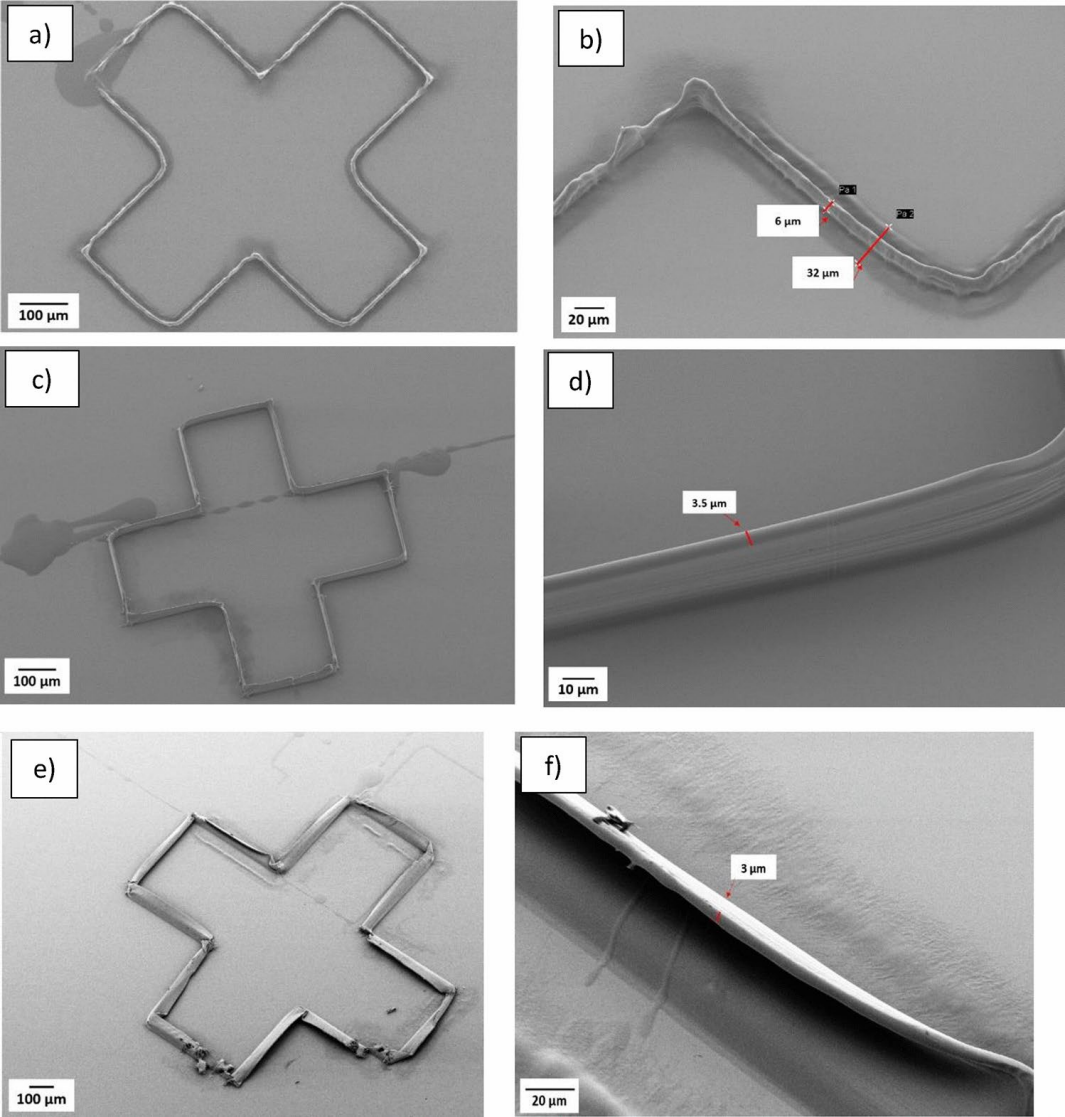
Ink with 12.5% CA (50 kDa) was used to study the effect of the moving speed of the substrate on the size and positioning of printed fibers: (**a**, **b**) 0.5 mm/s, (**c**, **d**) 1 mm/s, (**e**, **f**) 1.5 mm/s. The applied voltage was 700- 1000 V.

To investigate the possibility of decreasing the printing time, the printing behaviour for an increased movement speed was examined. In this study ink with 12.5% CA (50 kDa) and a voltage range of 700- 1000 V was used. The speed of the substrate movement in X and Y directions was investigated in the range of 0.5 mm/s to 2.5 mm/s, where 2,5 mm/s is more or less the maximum speed of the printer. At this speed the inaccuracy of the fiberplacement became too large to print the evaluation structure in Fig. 1(a) and hence the printing results for 2,5 mm/s are not presented. As images in Fig. 6(a- f) demonstrate, there is a relation between the speed and fiberwidth. By increasing the speed from 0.5 mm/s to 1 mm/s the width of the fiber reduced from almost 6 µm to 3.5 µm in the upper layers. This is in accordance with a constant ink flow rate and a more or less similar layer thickness. Somewhat surprisingly the same layer thickness of 1 µm has proven to provide the most stable printing for many layers (< 30 evaluated) in these experiments independent of printing speed (presented in more detail in the next section). As evidenced by Fig. 6(c), when the printing speed was set to 1.5 mm/s, a reduction in control of the fiber placement was observed, here the width of the printed fibers closely aligns with the nozzle inner diameter, which is 3 µm. Furthermore, results reveal that higher printing speed reduces the orthogonal spreading of the ink on the substrate for the first printed layer. As shown in Fig. 6(a, b) the width of the printed fiber in the first layer at 0.5 mm/s is measured to be 32 µm, exceeding the outer diameter of the nozzle (9 µm), but at 1 mm/s in Fig. 6(c, d), the width of the first printed layer is about 3 µm, matching the inner diameter of the nozzle. The non-continuous printing observed in Fig. 6(e, f) is partially due to the rather difficult printing pattern. The stage cannot keep a constant speed in the corners and this affects the printing control and placement. For printing of a fibrous network, a printing speed of 1 mm/s was selected. The printing speeds evaluated here are still low for applications in e.g. separation membranes and there is a need for developing printing equipment and strategies that allow for higher speeds.

### Printing fibrous network

**Fig. 7:**
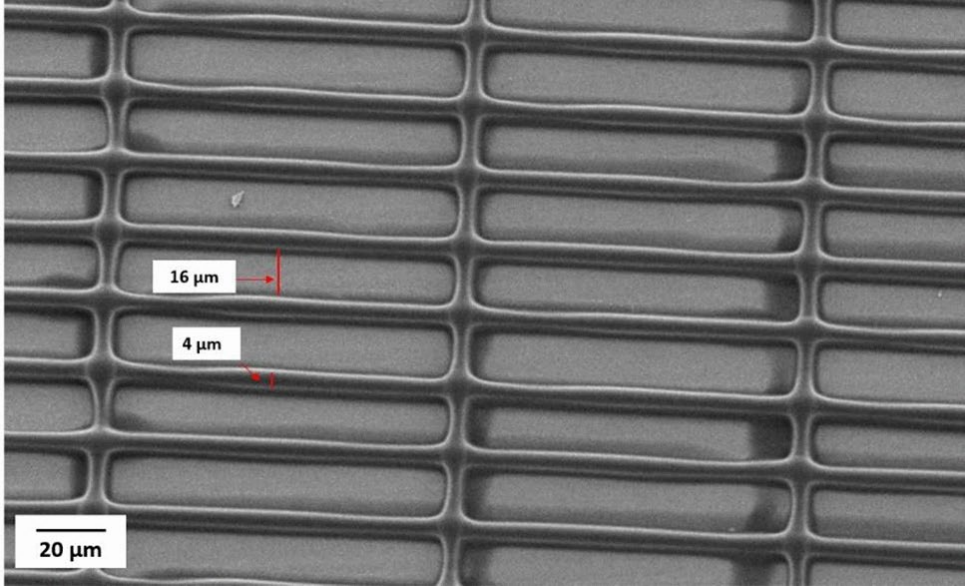
Printed structure with 20 µm pitch. The width of the fibers is equal to 4 µm and the inter-fiber distance is 16 µm.

One of the main objectives of this study was to investigate the possibility of printing fibers close to each other resembling the fibrous network used in separation membranes, in these experiments, ink containing 12.5% CA (50 kDa) with an applied voltage of 700- 1000 V have been used. For membrane printing the movement speed of the substrate in the X and Y directions is 1 mm/s. As given in the experimental section, the movement in the Z direction after printing each layer, the layer thickness, was selected to 1 µm. Before selecting a layer thickness of 1 µm, also 0.5 µm and 1.5 µm were examined. When the layer thickness was selected to 1.5 µm, the printing became unstable. At a layer thickness of 0.5 µm, the Taylor cone of the ejected ink became too close to the previous layers, which made it impossible to print fibers as well as get separate layers. Figure 7, demonstrates a successful printing of fibers with 4 µm width. In this case (Fig. 7) the 20 µm pitch results in an inter- fiber distance (between adjacent fibers) of 16 µm. The printing process was halted after about 10 layers of printing.

**Fig. 8:**
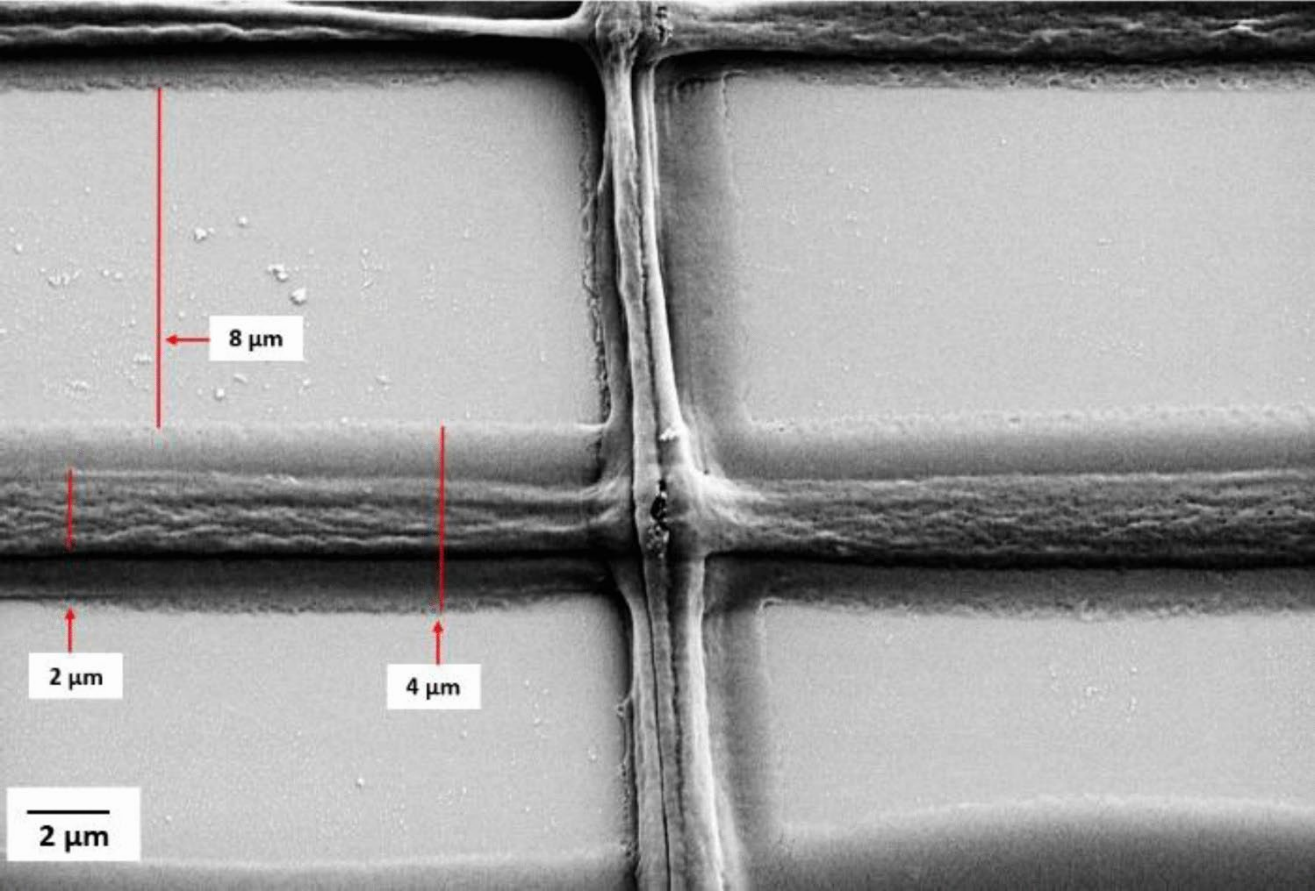
Printed fibers with 12 µm pitch and inner fiber distance of 8 µm. The width of the fiber in the first layer is 4 µm and it is reduced to almost 2 µm in the upper layers.

**Fig. 9:**
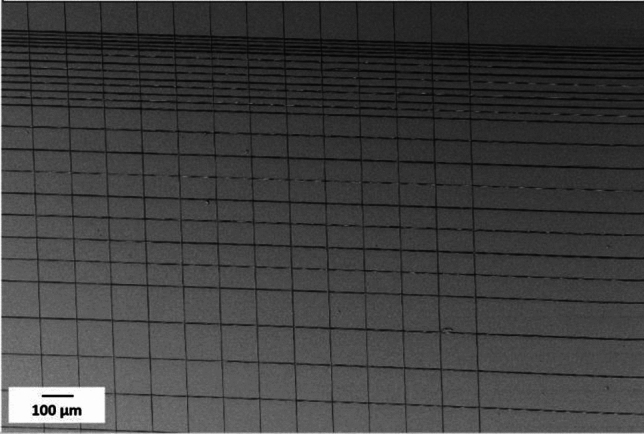
Pattern with different pitches from 100 µm to 15 µm.

Subsequent experiments to explore the feasibility of printing precise structures were made at reduced pitch values of 15 and 12 µm. Figure 8 at a pitch of 12 µm, shows that the inter- fiber distance was equal to 8 µm, and the initial printed fiber measured 4 µm. When printing upper layers, the orthogonal spreading of the ink between the layers is less than that of the substrate and first printed layer. Subsequently, the width of the fibers notably reduces to nearly 2 µm. Figure 9 presents an overview of the printed structure comprising five layers, after five layers the printing had been halted. The test structures also revealed the threshold for printing separated fibers. Reducing the pitch further than 10 µm in the first layer results in fiber adhesion.

**Fig. 10:**
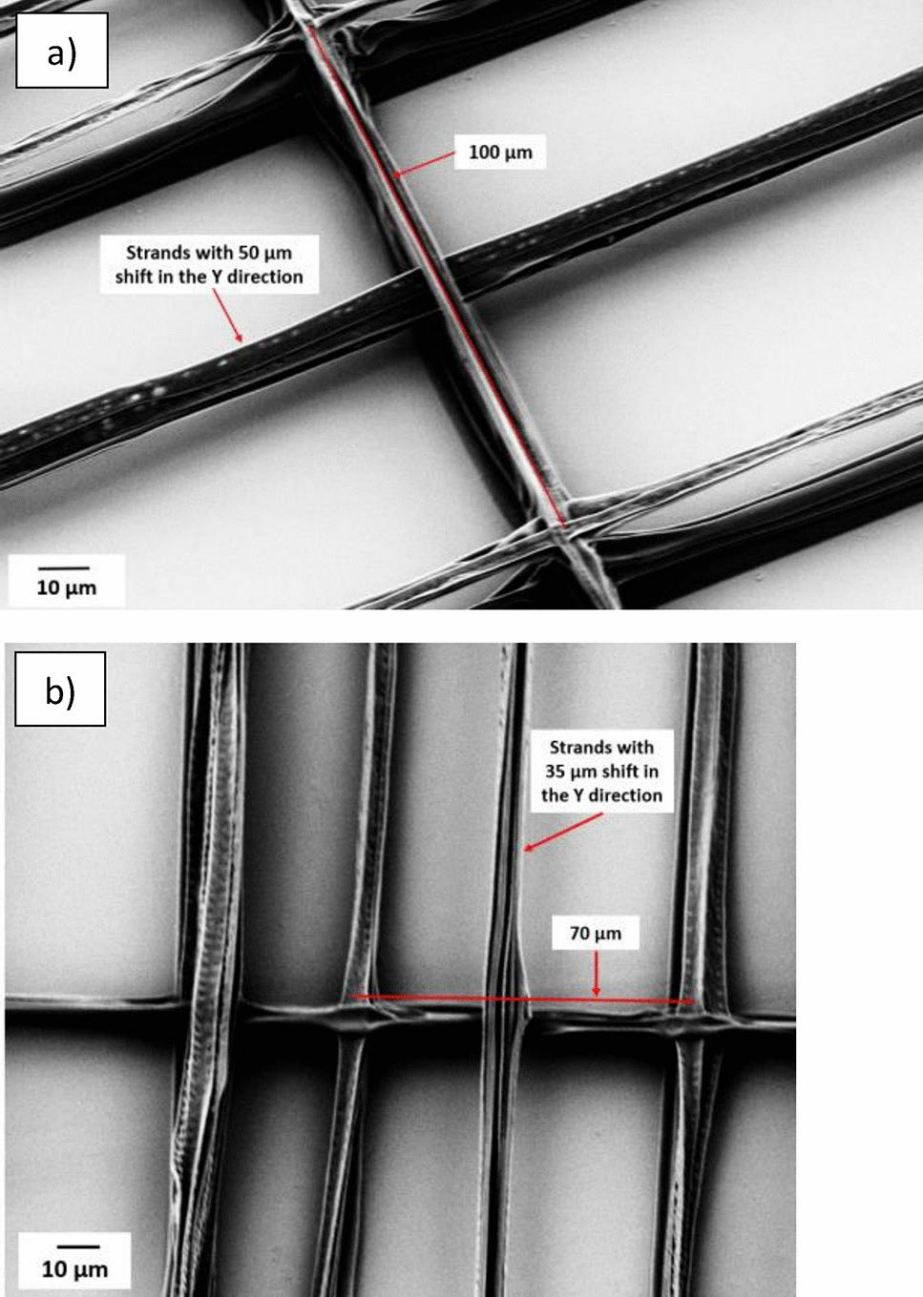
Printing free-hanging fibers (pattern 2(d):) (**a**) supporting pitches are 100 µm (inter-fiber distance 96 µm), (**b**) Supporting pitch reduced to 70 µm (with inter-fiber distance of 67 µm).

The pattern in Fig. 1 (d) was used to assess the feasibility of printing free-hanging fibers between supports. In this test, the first five layers are represented by the black lines in Fig. 1 (d), and three layers on top are shown with red lines in that pattern. Two different pitch distances were printed in the first five layers, 100 µm, and 70 µm. Upon reaching the sixth layer, a 50 µm shift in the fiber’s position in the X-direction and shifts of 35 or 50 µm in the Y-direction were introduced in relation to the first five printed layers, to place the upper layer fibers in the middle of the lower layer fibers. Figure 10(a, b) indicates successful printing of the free-hanging fibers with a support separation of 100 µm and 70 µm. Results indicate that when the pitches between supports in the underlying layer are set to at least 100 µm and 70 µm (Fig. 10 (a, b)), free-hanging fibers are positioned as designed, with placement error less than 2 µm.

**Fig. 11:**
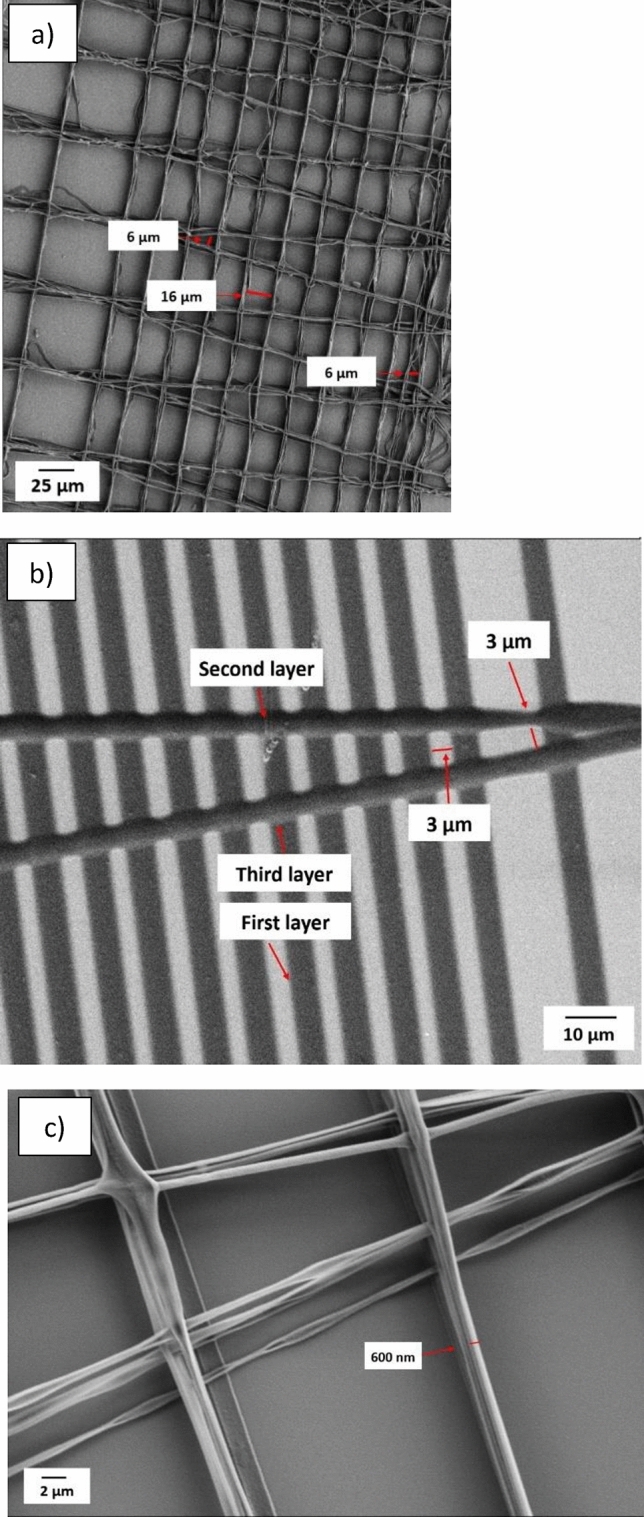
(**a**) Pattern 1 (e), structure with the smallest pitch of 10 µm with more than 21 layers, (**b**) three first layers of pattern 2 (f), with the smallest pitch of 5 µm and inter-fiber distance of 3 µm, (**c**) free-hanging fibers within sub-µm diameter.

Patterns in Fig. 1(e, f) were printed to estimate the minimum inter- fiber distance when, for instance, fabricating a scaffold-like structure. The pattern in Fig. 1(e), was printed in more than 21 layers, and the printed structure is shown in Fig. 11(a). The inter- fiber distances are about 16 µm and 6 µm when the pitches are set to 20 µm and 10 µm. Figure 11(b) shows a structure printed with three initial layers (pattern 1(f)), where the smallest inter- fiber distance is 3 µm for a pitch of 5 µm and an inter- fiber distance of 3 µm can be identified also in the perpendicular direction. Note that also solid structure is printed with a pitch of less than 3 µm for the first time using electroprinting, which might be of particular interest in certain designs. Moreover, Fig. 11 (c) illustrates that, when printing free-hanging fibers, it is possible to produce fibers with dimensions in the sub-µm range. The combination of electrostatic and pulling forces due to chain entanglement appears to stretch the fibers in analogy with electrospinning.

## Conclusion

This study demonstrates the potential of electroprinting for the precise fabrication and placement of cellulose acetate (CA) fibers, with potential in separation and bio-applications. By optimizing parameters such as nozzle-to-collector distance, polymer concentration, and applied voltage, sub-micron fiber diameters and inter- fiber distances as small as a few µm were achieved. Free-hanging fibers were also successfully printed, showcasing the versatility of this technique. A key innovation in this work is the intentional control over fiber positioning, enabling the fabrication of precise, well-defined structures—a significant improvement over traditional electrospinning methods, where fiber placement is often random. Our hypothesis that electroprinting at small nozzle-to-collector distances can be used to achieve highly detailed fiber arrangements was confirmed by these results. The placement control achieved is an order of magnitude better than what has been presented previously. While the process achieved high-resolution structures, low printing speed remains a challenge for scalability. Future research should focus on improving printing speed which calls for the development of both equipment and printing strategies.

## Data Availability

The datasets used and/or analysed during the current study available from the corresponding author on reasonable request.
